# Notes and News

**Published:** 1903-01-24

**Authors:** 


### Notes and News.

The Chelsea Hospital for Women has received from H. M. E. a further munificent donation of £2,000.

The International Congress of Life Assurance Medical Officers will hold their third Congress in Paris in May.

Dr. Symes Thompson will deliver four lectures on Digestion at the Gresham College on January 27th, 28th, 29th and 30th at six o'clock.

The study of biology at Cambridge should receive considerable impetus through the bequest of between £50,000 and £60,000 by the late Mr. Frederick J. Luck for the purposes of biological study and research.

In Paris a bureau of medical information has been established. It was formed through the initiative of M. Brouardel, assisted by De Blondel. The Municipal Council have made a grant for the support of the scheme, which will largely assist foreign and provincial persons desirous of studying in Paris.

Mr. T. Sutton Timmis is devoting £10,000 to the investigation of cancer, to be carried out at the Liverpool Royal Infirmary and the Liverpool University College. The German Cancer Research Committee are gathering up information throughout their country, and in Spain a large amount of information has already been collected by a similar organisation.

The treatment of consumption is by no means at a standstill. Germany was practically the first country to establish sanatoria inland, and now it is about to lead the way by an experiment to create floating sanatoria to cruise in suitable waters with patients. This of course applies to floating sanatoria on a large scale. An experiment on the same lines has been witnessed lately at Westcliff-on-Sea, off whose shore a motor hospital ship for consumptives has been lying. This innovation comes from America, and is reported as being very comfortable.

Dr. Heywood Smith has been elected president of the British Gynæcological Society.

The accounts of the Hospital Saturday Fund for the past year show an increase of £1,400.

A Jewish benefactor has contributed £10,000 to the London Hospital for the erection of a Jewish ward and kitchen.

A most enjoyable entertainment, arranged by Mrs. Humphreys, for the in-patients of the Cancer Hospital, Fulham Road, London, S.W., took place last Thursday evening.

We have been asked to state that fancy dress will be optional at the Grand Advertisement and Poudré Ball, to be held in aid of the Metropolitan Hospital on February 10th, at the Royal Palace Hotel, Kensington.

An anonymous donor has offered to provide a nurses' home, costing £8,000, for the Glasgow Samaritan Hospital for Women. The question of site is causing a little difficulty, which probably will soon be overcome.

The State Board of Charities of New York has been approached by a new organisation for a certificate of corporation. The establishment calls itself the "Lorenz Orthopædic Charity Hospital of New York City," and has for its object the treatment of deformities by the Lorenz method. The plea of "bloodless surgery" will no doubt appeal to numbers of persons who regard surgery associated with the knife with alarm or disapproval.

Frankfort is about to devote a good deal of attention to the improvement of post-graduate medical study. An Academy of Practical Medicine has been formed, facilities have been given by the donation of a private benefactor to establish suitable buildings, and all the medical institutions in Frankfort are to be affiliated with the Academy. The Municipality is taking its share of the work by the enlargement of the City Hospital.

The medical world will be interested in the announcement that the admirable "Index Medicus" is to be revived. The Carnegie Institution in Washington is devoting $10,000 a year to the work, which is to be purchased at $5, or 25s. per annum. It is a matter of congratulation to the Carnegie Institution that they have had the public interests so wisely and generously at heart. Works such as the "Index Medicus" should not be left as a matter of private or business venture, and it is a worthy object for the generosity of public benefactors such as Mr. Carnegie. The new Index will be conducted by Dr. Fletcher, of the American Army Medical Museum, and Dr. Fielding Garrison. The subject part of the work is subdivided and classified afer the manner adopted for the Index Catalogue of the library of the Surgeon-General's office. The titles in the cases of the less familiar languages are translated, as are some of the articles themselves. The numbers commencing in January will be issued at the commencement of each subsequent month.

